# P300 latency and amplitude in Alzheimer's disease: a systematic review

**DOI:** 10.1590/S1808-86942012000400023

**Published:** 2015-10-20

**Authors:** Renata Valle Pedroso, Francisco J. Fraga, Danilla Icassatti Corazza, Carla Andrezza Almeida Andreatto, Flávia Gomes de Melo Coelho, José Luiz Riani Costa, Ruth Ferreira Santos-Galduróz

**Affiliations:** 1 B.A. in Physical Education - Julio de Mesquita Filho Paulista State University (UNESP); MSc student in Human Motility Sciences - Julio de Mesquita Filho Paulista State University (UNESP); 2 PhD from the Aeronautics Institute of Technology (ITA) (III Adjunct Professor - Federal University of ABC - UFABC); 3 MSc in Human Motility Sciences - Julio de Mesquita Filho Paulista State University (UNESP); PhD student in Human Motility Sciences - Julio de Mesquita Filho Paulista State University (UNESP); 4 B.A. in Psychology - Uniararas (MSc in Human Motility Sciences - Julio de Mesquita Filho Paulista State University (UNESP); 5 PhD in Collective Health - UNICAMP (University of Campinas); Assistant Professor * UNESP); 6 PhD in Psychobiology - Federal University of São Paulo - UNIFESP; Adjunct Professor III - Federal University of ABC

**Keywords:** aging, alzheimer disease, cognition, event-related potentials, p300

## Abstract

The P300 plays a key role as a method for monitoring and evaluating dementia, including Alzheimer's disease.

**Objective**: The goal of this study was to search for articles which analyzed P300 latency and amplitude values in Alzheimer's disease.

**Methods**: We searched in the following databases: Web of Science, Pub Med, Psyc Info, Medline, Biological Abstracts and Scielo using the following keywords: speed of information processing, processing speed, information processing, aged, older, elderly, older people, alzheimer dementia, alzheimer disease, Alzheimer and cross-references of selected articles.

**Results**: We found eight studies matching the inclusion criteria. These studies showed that there is a consensus on a P300 latency increase of elderly patients with Alzheimer's disease compared with subjects without the disease. However, it appears that, with respect to the P300 amplitude, there is still no consensus; however, it may be related to different methodological variables adopted in the reviewed studies.

**Conclusion**: There is a need to standardize the variables involved in P300 measurement for senior citizens with Alzheimer's disease in order to be able to compare P300 latency and amplitude values for this population.

## INTRODUCTION

Event-related evoked potentials, also known as cognitive evoked responses - especially the P300, play a fundamental role as a method for dementia assessment and monitoring, Alzheimer's disease (AD) included^1^, ^2^, ^3^, ^4^.

The auditory P300 is characterized by a large amplitude wave, which is generated by the expectation of discrimination (in attention of) concerning a so-called rare stimulus (target), in opposition to a frequent stimulus (standard), and it appears approximately at 300 ms after the stimulus onset, varying to more than 400 ms, according to age and/or dementia^5^.

This is an important tool which assesses information processing time^6^, ^7^. In general, previous studies reported an increase in latency and reduction of the P300 amplitude in AD patients when compared to youngsters, elderly, or even patients with mild cognitive involvement^2^, ^8^, ^9^, ^10^, ^11^. Nevertheless, some of these studies leave *gaps* as to finding possible auditory involvement which may impact the results, as well as the confirmation of a clinical diagnosis of AD by using official criteria from international institutions (ICD, DSM, NINCDS/ADRDA), having in mind that many of the studies are very old.

Some papers went more in-depth and investigated the possible links between Alzheimer's and the lower performance (i.e., higher latency) in the P300 test, and the results found show a strong relation between them, such as: (1) The P300 is associated with other cognitive variables which are also impaired in patients with Alzheimer's, such as memory and cognition^12^, ^13^, ^14^ (2) Brain areas, such as the central-parietal cortex, frontal cortex and the hypocampus, generate the P300 wave, which are structures usually affected in patients with AD^4^, ^15^, ^16^, ^17^; (3) When involved, the cholinergic system, increases P300 latency, and AD impacts such variable^2^, ^18^, ^19^.

Thus, hereby we stress the importance of reviewing scientific papers which investigate the relationship between the P300 and Alzheimer's disease, since variables such as latency and amplitude, especially the former, may provide valuable information concerning information processing, which is impaired in AD. Having such reality in mind, the goal of the present study was to analyze P300 latency and amplitude in elderly with AD, in studies published in the literature, by means of searches in data bases and, afterwards, by crossed references in selected papers, such as the result of this search.

Our study may serve as support for healthcare professionals and suggests a reflection regarding the variables involved in the P300 assessment method, which has been utilized in studies with elderly with Alzheimer's disease, a population with impaired cognition, with difficulties of attention and task understanding.

## METHODS

The methodological process in this study was based on a systematic literature review, guided by bibliographic searches in the following databases: *Web of Science, Scopus, PsycINFO, Medline and Biological Abstracts*, Scielo. These databases were chosen because they specifically approach topics associated with health. Boolean operators and the keywords utilized were: P300 OR *electrophysiological* OR *evoked auditory cognitive potentials* OR *evoked potential* OR *auditory evoked potentials) AND (Alzheimer dementia* OR *Alzheimer disease* OR *Alzheimer)* NOT (*visual)*. There were no restrictions concerning the publication date of the papers. Besides the search in the databases, we also carried out a manual search in the reference lists of the selected papers.

The search for papers started in April of 2011, and we used the following inclusion criteria: (1) Alzheimer's Disease diagnosed according to criteria from official international agencies (ICD, DSM, NINCDS/ADRDA); (2) A sample made up of individuals who did not have auditory impairment; (3) Having an auditory P300; (4) Studies published in English and Portuguese; (5) Controlled studies with cognitively intact elderly. Those papers which do not meet these inclusion criteria were taken off this review.

## RESULTS

Our literature search resulted in 744 papers. In a first filtering, by reading the title, we noticed that 581 papers were unrelated to the topic, and there were 163 papers left. Of the papers which were excluded, 146 assessed patients with AD, 277 were not associated with the P300, 25 investigated the effect of substances, 15 were studies with animals and 118 were not associated either with the P300 or AD.

In the second sorting, reading the papers' summaries, we noticed that 108 papers were unrelated to the topic, and only 55 papers remained to be read entirely. Of the excluded papers, three were review papers, 24 did not assess patients with Ad, 47 were unrelated to P300, 15 were unrelated to the auditory P300, two were not controlled studies and 17 did not evaluate the relationship between the P300 and Alzheimer's disease.

By means of the last sorting, and after reading the entire papers, we selected the eight papers which fit our study. The papers excluded in this final stage did not match our study's inclusion criteria: 19 did not assess the auditory P300, 13 did not rule out auditory involvement, 10 were not diagnosed with AD according to the international agencies, five papers were not in English or Portuguese.

We describe below the eight studies selected for this systematic review:

1. Caravaglios et al.^20^ assessed P300 latency of 21 elderly with AD and had 16 healthy elderly as the control group. There were significant differences between the groups, in such a way that the AD group had a higher latency recorded from Pz, Fz and Cz leads when compared to the control group. We did not analyze P300 amplitude.
2. O'Mahony et al.^21^ also found significant differences in P300 latency when they compared senior citizens with AD (440.6 ± 65.2 ms) and 20 elderly from the control group (336.4 ± 36.8 ms). We did not analyze P300 amplitude.
3. Lai et al.^22^ assessed P300 latency and amplitude in Pz from three different groups: AD group (n = 20), group with mild cognitive impairment (n = 18), and the control group (n = 14). Besides the P300, they also had the subjects answer some neuropsychological questionnaires in order to assess attention, recent memory and language. There was a significant P300 latency difference in Pz among the groups, in such a way that the AD group had a higher latency than the group with cognitive involvement, which was higher than that of the control group. There was no significant latency difference in Fz and Cz. There was also no P300 amplitude difference among the three groups.
4. Yamaguchi et al.^23^ compared P300 latency and amplitude between the group with AD (n = 16), a group with vascular dementia (VD) (n = 16) and the control group (n = 14) and they used the paradigm of three types of stimuli to assess the P300: besides the rare and frequent stimuli they also added distracting stimuli (taken from movies for children).

Comparing P300 latency and amplitude for rare sounds: The AD and VD groups had a higher latency when compared to the control group; however, there were no differences between the dementia groups. There was a significant difference in P300 amplitude, in such a way that the AD and VD groups had lower amplitude when compared to the control group; nevertheless, there were no differences between the dementia groups.

Comparison of P300 latency and amplitude for distracting sounds: there was a significant difference in the P300 difference in such a way that the VD group was significantly higher than the AD and control groups. The AD group did not have a significant difference when compared to the control group. There was also a significant difference in the P300 amplitude, in which the VD group was significantly lower than the AD and controls. The AD group did not show significant differences vis-à -vis the control group.

5. Golob & Starr^10^ compared 10 senior citizens with Ad and 12 elderly in the control group as to P300 latency and amplitude. There was a statistically significant difference in P300 latency in Cz between the groups, in such a way that the AD group was higher than the control group. There was no P300 amplitude difference.
6. Bennys et al.^24^ compared P300 from 30 elderly with AD, 20 elderly with mild cognitive involvement and 10 control elderly. Moreover, the following neuropsychiatric testes were employed: Mini-mental, *Grober-Buscke, Trail Making Test*, Frontal Assessment Battery of tests. Although the paper did not show the exact values regarding P300, the authors showed significant differences in P300 latency among the three groups, in such a way that the AD group was higher than the group with mild cognitive involvement, which was higher than the control group. There was a significant difference also on P300 amplitude, in which the AD group had lower amplitude than the group with mild cognitive impairment and the control group. There was no difference between the control group and that with mild cognitive impairment as far as amplitude is concerned.
  The next two papers studied the P300 subcomponents, called P3a and P3b. P3a is associated with a passive response (unconscious) to a rare and new stimulus, in other words, the response not associated with attention. P3b is associated to the task of conscientious detection of a rare stimulus, which requires much more from attention and work memory^7^. P3b is what we call the P300 in other stimuli; therefore will be analyzed especially the values regarding P3b^25^.
7. Juckel et al.^26^ assessed P3a and P3b from the AD (n = 18) and Control groups (n = 18). The AD Group had a higher P3a latency than the control group, but it did not show amplitude differences. There were no significant differences between the groups in P3b latency; nonetheless, there was a difference in P3b amplitude - the AD group had a lower result than that of the control group.
8. Frodl et al.^3^ compared the P3a and P3b subcomponents of three groups: AD group, group with mild cognitive impairment and the control group of elderly. Besides these analyses, they also employed tests such as Verbal Fluency, Boston Naming Test, Free Word Remembering Test, Constructive Praxia and Word Recognition Test.

The P3a latency test was significantly higher in the AD group, when compared to the control group. The P3b amplitude was significantly lower in the AD group when compared to the controls. There were no differences when the AD group and that with mild cognitive involvement were compared.

In order to further explore the data, the authors also made an analysis in which AD individuals were broken down into two groups: mild and moderate, and they carried out another comparison with the mild cognitive involvement group. The results showed that the P3b amplitude was significantly higher in the group with mild cognitive involvement when compared with the elderly with mild and moderate AD.

Chart 1 presents the characteristics of the P300 assessment method of the reviewed studies and Chart 2 depicts P300 amplitude and latency results in the same studies.

**Chart 1:** P300 assessment method characteristics in elderly with Alzheimer's utilized in the reviews studies.

Paper	N^o^	Leads	Stimulus	Probability	Sti. duration	Sti. Interval.	Sti. total	Task	Instrument	dB
Caravaglios et al., 2008	1	Fz, Cz and Pz	Pattern =1000 Hz Target =2000 Hz	Pattern: 80%Target: 20%	60 ms	3.5 to 5.5 s	1024	Press button with the dominant hand	Ear phones	75
Mahony et al., 1996	2	Fz, Cz and Pz	Pattern =1000 Hz Target =2000Hz	Pattern: 80%Target: 15%	50 ms	—	—	Raise right hand index finger	Ear phones	60
Lai et al., 2010	3	Fz, Cz and Pz	Pattern =1000 Hz Target =2000Hz	Pattern: 84.62%Target: 15.38%	50 ms	1 to 2 s	325	Press button with right index finger	Ear phones	80
Yamaguchi et al., 2000	4	Cz and Pz	Pattern =1000 Hz Target =2000 HzEnvironmental sounds	Pattern: 65%Target: 20%background sounds: 15%	Pattern e Target: 100 msbackground sounds: 200 ms	1 to 1.3 s	400	Press button	Ear phones	60*
Golob & Star, 2000	5	Fz Cz Pz C3 C4 T3 T4	Pattern = 1000 Hz Target = 2000 Hz	Pattern: 80%Target: 20%	100 ms	—	300	Press button with dominant hand	Speaker at 0.75 meters	70
Bennys et al., 2007	6	Fz, Cz and Pz	Pattern = 1000 Hz Target = 2000 Hz	Pattern: 80%Target: 20%	100 ms	1125 ms	150	Silent counting	Ear phones	60
Juckel et al., 2008	7	32channels	Pattern = 500 Hz Target = 1000 Hz	Pattern: 80%Target: 20%	40 ms	1.5 s	500	Press button	Ear phones	—
Frodl et al., 2002	8	29channels	Pattern = 500 Hz Target = 1000 Hz	Pattern: 80%Target: 20%	40 ms		500	Press button with dominant hand	Ear phones	80

* 60 dBs above each individual's threshold; ms: milliseconds; s: seconds; μV: microvolt; Hz: Hertz.

**Chart 2:** P300 latency and amplitude values in Alzheimer's disease.

Paper	N°	Characterization	Latency (ms)	Amplitude (μV)
			In Fz:	
		AD: n = 21 (mean age: 74.9 ± 7.4 years)	AD = 448.5	
			CG = 390.2	
			In Pz:	
Caravaglios et al., 2008	1		AD = 462.0	---------
			CG = 387.7	
		CONTROL: n = 16 (mean age: 74.0 ± 8.7 years)	In Fz:	
			AD = 458.0	
			CG = 391.3	
Mahony et al., 1996	2	AD: n = 18 (mean age: 74.5 ± 4.3 years)	AD = 440.6 ± 65.2	
		CONTROL: n = 20 (mean age: 72.7 ± 4.7 years)	CG = 336.4 ± 36.8	
Lai et al., 2010	3	AD: n = 20 (mean age: 71.04 ± 6.52 years)	In Pz:	---------
		MCI: n = 18 (mean age: 68.00 ± 8.70 years)	AD = 443.72 ± 2.81	
		CONTROL: n = 14 (mean age: 64.79 ± 7.75 years)	MCI = 418.78 ± 40.14	
			CG = 390.14 ± 27.23	
		AD: n = 16 (mean age: 68.5 ± 8.0 years)	AD = 454.3 ± 86.7	AD = 45.8 ± 4.3
Yamaguchi et al., 2000	4	VD: n = 16 (mean age: 68.9 ± 7.3 years)	VD = 462.8 ± 89.5	VD = 4.8 ± 3.2
		CONTROL: n = 16 (mean age: 69.6 ± 9.3 years)	CG = 361.7 ± 47.0	CG = 8.5 ± 4.0
		AD: n = 10 (mean age: 72.0 ± 3.1 years)	In Cz:	In Cz:
Golob & Star, 2000	5		AD = 383.4	AD = 3.7
		CONTROL: n = 12 (mean age: 66.3 ± 1.6 years)	CG = 333.9	CG = 6.4
		AD: n = 30 (mean age: 70.9 ± 6.8 years)	AD > MCI > CG	AD < MCI andCG
Bennys et al., 2007	6	MCI: n = 20 (mean age: 64.4 ± 7.6 years)	No values	No values
		CONTROL: n = 10 (mean age: 61.6 ± 6.4 years)		
			Latency P3a:	Amp. P3a:
		AD: n = 18 (mean age: 66.7.9 ± 10.2 years)	AD = 358.6 ± 41.6	AD = 2.6 ± 2.1
Juckel et al., 2008	7		CG = 325.9 ± 34.7	CG = 3.4 ± 2.9
			Latency P3b:	Amp. P3b:
		CONTROL: n = 16 (mean age: 63.8 ± 11.1 years)	AD = 339.4 ± 34.5	AD = 3.6 ± 2.1
			CG = 353.6 ± 28.8	CG = 5.7 ± 2.3
			Latency P3a:	Amp. P3a:
		AD: n = 30 (mean age: 69.9 ± 10.3 years)	AD = 369 ± 59	AD = 2.8 ± 1.7
			MCI: 352 ± 37	MCI: 2.7 ± 1.8
Frodl et al., 2002	8		CG = 327 ± 42	CG = 3.2 ± 2.5
		MCI: n = 26 (mean age: 66.2 ± 11.3 years)	Latency P3b:	Amp. P3b:
			AD = 362 ± 37	AD = 3.3 ± 1.9
		CONTROL: n = 26 (mean age: 64.9 ± 10.9 years)	MCI: 352 ± 37	MCI: 5.2 ± 2.1
			CG = 349 ± 32	CG = 5.3 ± 2.3

AD: Alzheimer's disease; VD: Vascular Dementia; MCI: mild cognitive impairment; CG: Control Group; ms: milliseconds; μV: microvolt.

## DISCUSSION

Upon analyzing the eight papers included in the present study, we noticed that four of them compared possible P300 differences between elderly with AD and those in the control group. Three papers compared elderly with AD, those with mild cognitive impairment and the control elderly. Only one study compared the P300 of elderly with AD, vascular dementia and elderly from the control group.

All the papers inserted in this review found significant differences in the P300 latency of elderly with AD and their control counterparts. Analyzing only the mean latency values of these studies, we can see that the group with AD varied between 358 ms^24^ and 458 ms^20^, depicting a 100ms margin between the lower and the higher values found in the studies. Young individuals tended to have P300 latencies around 300 ms^27^, Thus, 100 ms is a very large information processing variation time, since it represents one third of the information processing time in youngsters.

The studies showed a lower variation between the mean P300 latency values among the control elderly, since it varied between 325 ms^26^ and 391 ms^20^.

Maybe the major P300 latency variation in AD elderly found in the studies is because they had patients in different stages of dementia. O'Mahony et al.^21^ assessed AD patients with mild to moderate impairment, and despite not showing the mean scores in the Mini-test of the Mental Status (MEEM), the values from each individual varied from 9 points (severe involvement, according to Folstein^28^) to 27 points (questionable to mild involvement, according to Folstein et al.^28^), which shows that the patients were in varied levels of involvement^29^, ^30^. Thus, further studies are required to check P300 latency values in AD patients in the different stages of the disease.

The mean P300 amplitude values were assessed in only six of the eight reviewed studies and only four found significant differences between AD elderly and their control counterparts^3^, ^23^, ^24^, ^26^, showing that the P300 amplitude may not be an important different aspect between cognitively preserved elderly and those with AD. Some studies show that the P300 amplitude is associated with attention, and reduced amplitudes would be associated to a low level of attention^31^; therefore, maybe the variables involved in the P300 assessment method which were used in the study could have impacted the results.

The P300 assessment methodology is common to all the studies; however, the variables adopted in each of the them vary substantially, especially in regards of intensity, duration and type of acoustic stimulus, interval between stimuli, and even the task they must perform upon perceiving the stimulus. Thus, it is clear the importance of discussing these variables, since such heterogeneity makes it difficult to compare the values between papers.

The frequency of stimuli is one of the variables which did not change so much in the studies, since there is a large array of papers in the literature suggesting the use of 1000 Hz for standard acoustic stimuli; and 2000 Hz for uncommon auditory stimuli^32^, ^33^, ^34^, ^35^, ^36^. Notwithstanding, the studies from Juckel et al.^26^ and Frodl et al.^3^ used the same protocol which adopted the frequencies of 500 Hz and 1000 Hz for frequent and unusual stimuli, respectively. It is necessary to exert caution when using the lower frequency signals, since there is evidence that the P300 amplitude is reduced and its increased latency when low frequency sound stimuli are used in comparison to the use of higher frequency sounds^37^.

The total number of stimuli varied between 150^24^ and 1024^20^. For such variable, there is still no consensus regarding the ideal number of stimuli to perform the task. Notwithstanding, when the number of repetitions exceeds one hundred, there were habituation and fatigue effects, which cause apparent P300 response changes^38^. Thus, having seen that AD patients have attention deficits and other cognitive involvements, one should adopt the lowest possible number of stimuli which do not compromise the results of the study.

The interval between the stimuli varied between 1 second^22^, ^23^ and 5.5 seconds^20^. Among the factors influencing habituation (i.e., the individual gets used even with the rare stimulus) in the P300 tests are the inter-stimuli intervals, the interval between target stimuli (rare) and the interval between groups of stimuli. Larger P300 amplitudes and lower habituation are associated with higher values for all these temporal parameters^39^.

The stimulus intensity varied between 60 and 80 dB. According to Spirduso^40^, stimulus intensity is one of the factors with impact information processing speed. Papanicolau et al.^41^ confirmed that P300 may vary in accordance to the stimulus intensity: a P300 latency variation was found among young adults submitted to two stimuli with different intensities.

Most of the studies used ear phones for the exam, except the one from Golob & Starr^10^, which used speakers at 0.75 meters from the individual assessed. Duarte et al.^42^ did not find P300 amplitude and latency differences when they compared tests using ear phones with speakers; however, the very paper stresses that all the subjects who participated in the study were adults without cognitive involvement; therefore, the data cannot be extrapolated for other populations.

The task complexity is also a factor which influences P300 values, and César & Munhoz^43^ found longer latencies in less complicated tasks (such as “silent counting”) when compared to more complex tasks (such as “raise one's hand).

In general, the task adopted in the papers part of this review seem to be more complex, it is necessary to “press the button” whenever the individual would hear the rare stimulus, except for Bennys et al.^24^, who chose to “count silently”. Notwithstanding, a more careful reflection is suggested when dealing with P300 assessment for elderly with Alzheimer's disease and difficulty in understanding tasks. When the patient makes a silent counting, it is difficult to “follow” the exam and check whether the patient really has his/her attention towards counting the stimuli. Now, a more complex task, including a motor component, provides further support to the examiner, who can count on the visual analysis of the test performance, in order to check whether the Alzheimer's patients are able to keep their attention during the test.

Having all the variables described in this review, we stress the importance of knowing the population which is being assessed, i.e. the Alzheimer's patients, since this particular group has numerous cognitive impairments which must be taken into account in order to choose the most adequate experimental variables for this population.

The lack of studies on this topic results in some questionings, such as which would be the most adequate variables to use the P300 for elderly with Alzheimer's? Therefore, we believe that further studies are needed to support a standardization of the variables involved in the P300 assessment method for patients with Alzheimer's disease.

## CONCLUSION

This systematic review showed that there is a consensus in relation to P300 latency increase in elderly with Alzheimer's disease when compared to those without it. Notwithstanding, we noticed that as far as the P300 amplitude is concerned, there is no consensus; however, this may be associated to the different variables adopted in the reviewed studies.

We also noticed that there is a need to standardize the variables involved in the P300 assessment method, so that it can be possible to compare the values obtained in each study. Regarding elderly with dementia, one must consider its limitations, disease signs and symptoms in order to choose the most adequate variables for the test. Moreover, it is suggested that studies should assess patients with AD separated by level of involvement, in other words, in mild to moderate stages of the disease, in order to better understand the relationship between information processing and Alzheimer's progression.
